# Enhanced passive mixing for paper microfluidics†

**DOI:** 10.1039/d1ra04916j

**Published:** 2021-07-26

**Authors:** Nurul Nadiah Hamidon, Gert IJ. Salentijn, Elisabeth Verpoorte

**Affiliations:** Pharmaceutical Analysis, Groningen Research Institute of Pharmacy, University of Groningen 9700 AD Groningen The Netherlands E.M.J.Verpoorte@rug.nl +31 50 363 75 82 +31 50 363 33 37; Faculty of Industrial Sciences and Technology, Universiti Malaysia Pahang 26300 Kuantan Malaysia; Laboratory of Organic Chemistry, Wageningen University and Research Stippeneng 4 6708 WE Wageningen The Netherlands

## Abstract

Imprecise control of fluid flows in paper-based devices is a major challenge in pushing the innovations in this area towards societal implementation. Assays on paper tend to have low reaction yield and reproducibility issues that lead to poor sensitivity and detection limits. Understanding and addressing these issues is key to improving the performance of paper-based devices. In this work, we use colorimetric analysis to observe the mixing behaviour of molecules from two parallel flow streams in unobstructed (on unpatterned paper) and constricted flow (through the gap of a patterned hourglass structure). The model system used for characterization of mixing involved the reaction of Fe^3+^ with SCN^−^ to form the coloured, soluble complex Fe(SCN)^2+^. At all tested concentrations (equal concentrations of 50.0 mM, 25.0 mM or 12.5 mM for KSCN and FeCl_3_ in each experiment), the reaction yield increases (higher colorimetric signal) and better mixing is obtained (lower relative standard deviation) as the gap of the flow constriction becomes smaller (4.69–0.32 mm). This indicates enhanced passive mixing of reagents. A transition window of gap widths exhibiting no mixing enhancement (about 2 mm) to gap widths exhibiting complete mixing (0.5 mm) is defined. The implementation of gap sizes that are smaller than 0.5 mm (below the transition window) for passive mixing is suggested as a good strategy to obtain complete mixing and reproducible reaction yields on paper. In addition, the hourglass structure was used to define the ratio of reagents to be mixed (2 : 1, 1 : 1 and 1 : 2 HCl–NaOH) by simply varying the width ratio of the input channels of the paper. This allows easy adaptation of the device to reaction stoichiometry.

## Abstract 2

Efficient passive mixing can be achieved by contricting the reagent flow using structures having narrow gaps.

## Graphical abstract

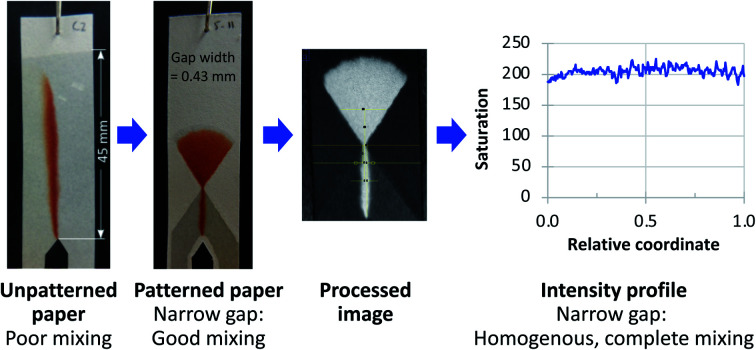

## 1 Introduction

There is a growing demand for portable analyses to quickly screen samples and products on-site for possible contaminants, disease markers, forensic evidence, and so on, thereby reducing the strain on centralized labs. The shift from lab-based to field-based analysis and detection not only increases information density but also allows results to be quickly disseminated.^1^ Much research has been dedicated to using paper as a substrate for the development of quick and affordable tests.^2–5^ The main reasons for this are that paper is an inexpensive and highly available material, and fluid transport occurs passively through capillary action.^6,7^ Finally, a plethora of patterning^8–10^ and modification^11–13^ strategies are at our disposal to produce functionalized paper microfluidic devices.

Traditionally, paper-based tests provided mainly qualitative, binary information, or semi-quantitative results at best. Recent advances in paper microfluidics have raised the expectations, with many different functionalities, such as pumps,^14–16^ timers,^17,18^ valves,^19–22^ gradient generators^23,24^ and filters^25^ being developed. The level of commercialization of paper-based devices, however, is still low, in spite of the above-mentioned merits. For now, producing devices for robust, quantitative testing remains a challenge.

The fact that the development of robust, accurate reaction-based assays on paper is so challenging stems from the often-inefficient interaction between chemical species in paper.^26^ When designing a paper-based device, not only the chemistry of the test itself, but also the transport behaviour of the dissolved reactants in the device needs to be considered. However, fluid flows on paper are difficult to control precisely, because paper is porous, fibrous, and flat, and swells when in contact with liquids.^27–30^ These characteristics make paper microfluidics very different from channel microfluidics, which is well-known for allowing excellent control over liquid flows.^31,32^ The aforementioned characteristics of paper are advantageous in that they give rise to capillary action, which allows passive movement of liquid towards unwetted regions in a paper substrate. However, the flow path that liquid follows is by definition obstructed by the tortuous network of cellulose fibres making up the paper. This leads to variation in local flow rates, which in turn leads to additional dispersion.^7,30^ Furthermore, the open nature of “channels” in paper devices leads to increased evaporation, which causes flow retardation and concentrating effects.^33^ The width of a paper microfluidic channel (several millimeters) is much greater than its thickness (generally below 200 μm), which is unfavourable for the efficient transverse transport of solutes (*i.e.* perpendicular to the direction of flow) that is necessary for mixing and chemical reactions. These cross-sectional dimensions differ significantly from channel microfluidics in which the channel height and width are usually in the same order of magnitude. Finally, cellulose fibres in paper will swell as a result of wetting, which influences the dynamics of capillary flow.^28^ All these factors can contribute to inhomogeneous distribution of chemical species on paper,^34^ making it difficult to control, let alone complete, a chemical reaction on a piece of paper. As a result, quantitative chemical analysis based on (colorimetric) chemical reactions becomes difficult at best.

Several studies have been carried out to overcome the adverse influence of imprecise fluid control on assay performance. Some of these have been dedicated to developing mixing strategies in order to increase assay reaction rate. More recently, researchers have investigated the behaviour of two different solutions when they meet in a paper microfluidic channel.^7,8,24^ Osborn *et al.* have shown that stacking two pieces of paper, each with its own source of liquid, causes analytes from the different sources to mix in the overlap region between the two layers, referred to as the “interdiffusion” zone. This vertical mixing mechanism was used to produce a flat Y-shaped mixer and diluter for paper-based devices.^8^ A similar stacking strategy has also been employed more recently by us to allow the fast exchange of solutes between two immiscible solvents in a counter-current configuration.^35^ Rezk *et al.* have demonstrated the use of acoustic waves for mixing liquids flowing side-by-side in a serpentine channel.^36^ While a 3D paper device, consisting stacks of several papers, combined with acoustic mixing appeared to be more efficient than a simple 2D paper device, such innovations can add undesirable complexity to the device in terms of the production process.

Recently, Urteaga *et al.* discussed the role of the structural properties of paper and flow velocity with respect to the extent of transverse dispersion of dissolved chemical species across the interface of two co-flowing liquids in a single layer of paper.^7^ This work revealed that the extent of dispersion is mainly dictated by properties of the porous substrate rather than the fluid velocity and species-specific diffusivity. The fact that chemical species in different solutions only mix partially when they flow side-by-side, even in a mm-wide channel, was exploited in order to design and generate chemical gradients on paper.^24^

To the best of our knowledge, there have to date been no reports of any single-layer, easily produced, equipment-free mixer that can be easily incorporated into 2D paper devices. Most of the existing on-paper mixers involve either additional equipment to supply an agitating force to achieve mixing in the porous paper network, or layering of paper to create overlapping solution zones and thus shortened diffusion distances. While effective, the latter approach could result in additional steps being required for device production.^26^ Thus, a simple mixing strategy is important for achieving devices that are truly user-friendly, easy-to-transport and affordable. Furthermore, a straightforward fabrication process not only reduces production costs but would encourages production scale-up initiatives as well, especially for small manufacturers. This in turn will stimulate the introduction of diverse diagnostic tools to meet the various needs of end-users.

In this work, a suitable geometric structure is introduced as a tool to promote passive mixing in paper-based devices. Co-flowing streams are generated and in-depth characterization of the solution behaviour at the interface between these streams is performed. This work focuses on the visual detection of mixing through the use of digital image colorimetric analysis, and thus aims to assess mixing and interaction from an experimental perspective, rather than a theoretical perspective. It builds on, and is highly complementary to, aforementioned recent literature on this subject, in which transverse dispersion in co-flowing systems is discussed,^7^ and its use applied to the generation of concentration gradients.^7,24^

## 2 Methodology

### 2.1 Materials and equipment

**Fig. 1:**
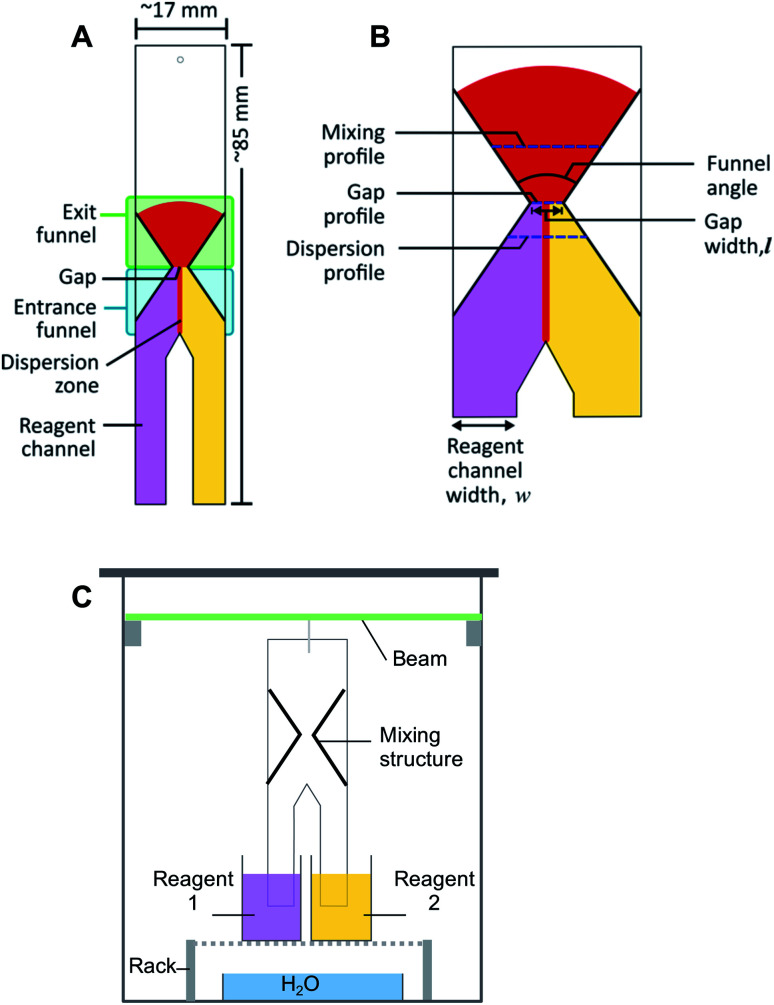
Schematic representations of (A) the paper structure proposed for the passive mixing of liquids on paper, (B) an expanded view of the hourglass structure, showing the design parameters and (C) the experimental setup. Experiments are conducted with the paper devices positioned vertically. Flow of solutions in all schematics is from the bottom to the top, where pink and yellow colours represent reagent solutions, and red the solution of reaction product.

Paper microfluidic devices were produced using Whatman Grade 1 chromatography paper (GE Healthcare Life Sciences, China), alkyl ketene dimer (AKD, 22-carbon chain, courtesy of Ashland Inc., Finland), trimethyl (tetradecyl) ammonium bromide (TTAB, Sigma Aldrich, India), an Isograph refillable plotter pen with 0.6 mm nib (Rotring, Germany), XY-plotter (v2.0, Makeblock, China) and oven (OMT, Sanyo Gallenkamp, U.K.), as described in previous work.^9^ Ferric chloride hexahydrate (FeCl_3_·6H_2_O; Merck, The Netherlands), potassium thiocyanate (KSCN; Fluka, The Netherlands), sodium hydroxide (NaOH; Sigma Aldrich, The Netherlands), hydrochloric acid (HCl; Acros, The Netherlands) and Phenol Red (Sigma Aldrich, The Netherlands) were used to prepare solutions for the colorimetric analysis. Demineralized water was used for making all of the solutions. A glass chamber with a 3D-printed suspended beam (polylactic acid, EasyFil filament with a diameter, *d* = 1.75 mm, Formfutura, The Netherlands), made with a fused-deposition modelling printer (Felix v.3, The Netherlands), was used for running experiments. Paper devices were suspended from this beam, with the ends of their reagent channels immersed in reagent solution reservoirs, see Fig. 1C. A microscope (Leica S8 APO, Germany) and digital camera (Canon EOS 700D, Japan and Olympus XZ-10, China) were used to acquire images. The image analysis was performed using the computer program, ImageJ.^39^ Microsoft Excel and the programming language, R, were used for data analysis and plotting.

### 2.2 Design of a passive mixer for paper microfluidics

The schematic diagram of the general structure proposed for the passive mixing of liquids on paper is depicted in Fig. 1A. The design consists of two ‘legs’ (reagent channels) that can be suspended vertically in different reagent solutions. Where these “legs” or channels meet, transport of solute between solutions can start to take place. Further downstream, an hour-glass shaped hydrophobic barrier is introduced, which collects all flowing liquid by means of an entrance funnel and guides it through a small orifice (gap). This latter feature was designed to enhance the degree of mixing between the two solutions. After the gap, the hydrophobic structure expands again to form an exit funnel. Different design parameters, including gap width (0.32–4.69 mm), funnel angles (67°, 30° and 15°), and the ratio between the width of the reagent channels (2 : 1, 1 : 1 and 1 : 2 HCl : NaOH) (Fig. 1B), were varied for different experiments.

### 2.3 Patterning of paper

Fabrication of the paper microfluidic devices was done *via* a previously reported procedure.^9^ In short, an aqueous suspension of AKD was loaded into a plotter pen cartridge, with the pen then being attached to the XY-plotter. Structures were designed in *Inkscape*, an open-source graphic editor; the *gcode* file it generated was sent to the XY-plotter software. The plotter was then used to draw the desired outline of the structure on the paper. Afterwards, the paper was heated in the oven at 80 °C for 15 min to allow melting, spreading and bonding of the AKD with the cellulose, thus producing hydrophobic structures. Paper which contained multiple test structures was then cut using a ruler to guide a scalpel along the drawn outline, producing individual pieces with dimension of approximately 17 × 85 mm (Fig. 1A). The patterned paper was then marked and wetted with water to inspect the mixing structure and the gap width using the microscope. An image was acquired with a ruler as size reference. An example of the image can be found in the ESI (Fig. S1†). ImageJ was then used to calculate the actual width of the gap.

### 2.4 Experimental procedure

The setup for experiments with passive mixing through the use of the hourglass-shaped structure is given in Fig. 1C. Paper strips patterned with the mixing structure were suspended in a glass chamber. Inside the chamber, a 3D-printed beam was placed, from which the strips could be suspended into reservoirs containing different reagents. Paper devices had two reagent channels or “legs”, as shown in Fig. 1A; these could be dipped into separate reagent-containing reservoirs (25 mL), as shown in Fig. 1C. The reagent channels served to transport the solutions from the reservoirs into the mixing device. A separate reservoir containing water was placed at the bottom of the chamber and a glass cover was placed on the chamber to close it in order to maintain the humidity in the chamber. The image of the paper was captured, using a digital camera, after the fluid front had travelled through the exit funnel. Photographs of the system were acquired while solutions were flowing to exclude any effects (*e.g.* flow retardation, variation of flow rate) brought about by the drying process. Effectively, this means that images were taken through the transparent, glass wall of the chamber.

To optimize the geometry of the hourglass structure, experiments were performed using FeCl_3_ in water (12.5–50.0 mM) as the first reagent and KSCN in water (12.5–50.0 mM) as the second reagent. The concentrations of the two reagents were always equal for any given experiment. The reaction between Fe^3+^ and SCN^−^ ions shown in eqn (1) (equilibrium constant, *K* = 105–146)^37,38^ was used as the test model for the colorimetric analysis. We used this reaction to assess how the yield and distribution of product could be improved by the implementation of the proposed mixing structure. The appearance of a red colour denotes that the reaction has taken place, which implies the prior presence of both Fe^3+^ and SCN^−^ ions at that particular location, *i.e.* mixing must have occurred.1
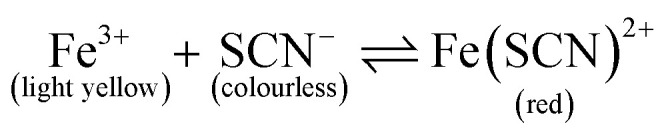


This reaction was chosen because the formation of product is heavily favoured, as indicated by the large equilibrium constant for this reaction. Mixing these two ions results in a single product, that afterwards will not undergo a chromatographic separation as it is wicked through the paper substrate. Furthermore, since the reaction product is strongly coloured, the reaction, and thus the mixing can easily be monitored. This is clearly different from a system where the mixing is done with two dye solutions, as they can ‘unmix’ by paper chromatographic separation. Initially, paper strips with just reagent channels were tested to assess the level of mixing in the absence of the hourglass structure. Next, paper strips with the hourglass structure were tested. As described in Section 2.2, devices with varying gap widths and varying funnel angles were tested for their mixing performance in these experiments. The width ratio between the reagent channels was kept constant at 1 : 1 KSCN–FeCl_3_ (*w* = 5–6 mm) for all these experiments. Experimental data for approximately 30 devices comprising various gap sizes were collected for each concentration tested.

An optimized design (again with the same paper width of ∼17 mm) with a gap width of 0.25–0.44 mm and an angle of 67° was then used to investigate the influence on mixing behaviour of varying the width ratio of the reagent channels (2 : 1, 1 : 1 and 1 : 2). The *w*_HCl_ and *w*_NaOH_ for 2 : 1, 1 : 1 and 1 : 2 are approx. 10 and 5 mm; 8 and 8 mm; and 5 and 10 mm, respectively. Different reagents were used for this characterization, namely a solution of HCl (10 mM) containing Phenol Red (2 mM) and a solution of NaOH (10 mM).

### 2.5 Image acquisition and analysis

The acquired images were transferred to the open-source software, ImageJ, in which regions of interest (ROIs; *i.e.* pixel-sampling area) were defined for the image analysis. The ROIs were defined in such a way that pixels along the mixing profile and at the dispersion profile (Fig. 1B) could be sampled to quantify the mixing efficiency and the width of the dispersion zone, respectively. The mixing profile was defined as the transversal line in the middle of the exit funnel. The dispersion profile was defined as the transversal line in the entrance funnel, located a quarter of the distance from the gap to the point where the two reagent channels come together. Control ROIs for the reagent solutions were included for each image. The image was then converted into the hue-saturation-brightness colour space (HSB stacks) and the saturation reading was recorded and analysed. The saturation reading was compared to that of pre-mixed solutions, that were allowed to wick into unpatterned paper. The detailed procedure for defining the ROI, conversion of images, and data treatment can be found in the Procedures section of the ESI.†

The quantification of mixing performance was assessed *via* two parameters: average saturation, and relative standard deviation (RSD). The first parameter is the saturation of the colour of the reaction product. Saturation is one of the three dimensions of the HSB colour space, and a value between 0 and 255, corresponding to the range between white and fully saturated, can be correlated to the amount of reaction product present. Therefore, a high saturation (*i.e.* high signal) means a high concentration of product. The second parameter is the RSD of the colour intensity profile across the exit funnel. Low RSD is obtained when reaction products are homogeneously distributed (*i.e.* uniform signal) across an area. A homogeneous saturation across an area directly corresponds to good mixing. The mixing model used in this experiment contains reactive substances, meaning that mixing increases the amount of product rather than re-distributing the fixed amount product as in non-reactive mixing. Thus, higher reaction yield (*i.e.* higher signal intensity) is also made possible by better mixing.

## 3 Results and discussion

### 3.1 Co-flow mixing without the hourglass structure

**Fig. 2:**
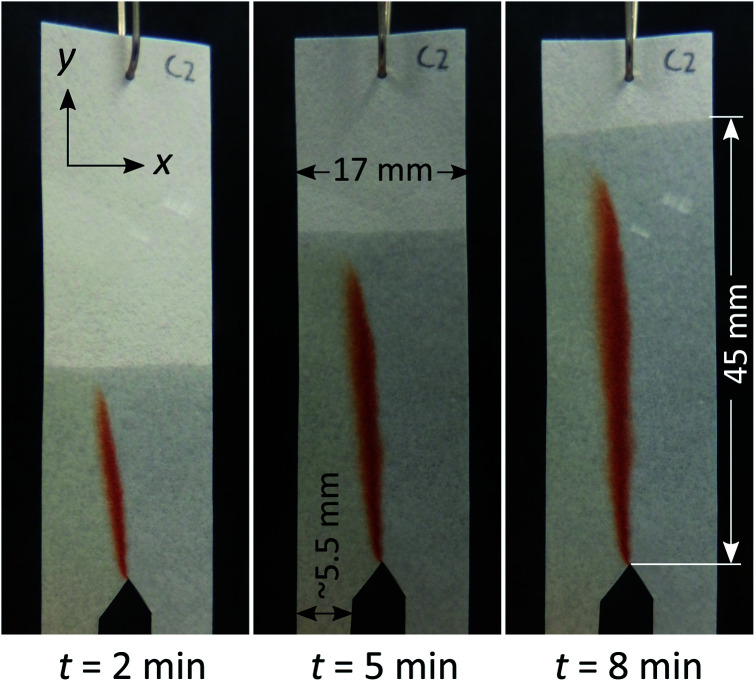
These photographs document what happens over time when two reagent solutions meet in the lower region of this paper strip. The solutions mix at the interface between them, with the subsequent formation of reaction product (red band) in a continuous co-flow. The reagent channels are separately suspended into reservoirs containing 50.0 mM FeCl_3_ (left side) and 50.0 mM KSCN (right side). The deviation of the dispersion zone from the vertical centre of the paper is simply caused by the variation in flow rate of the two solutions. This is very common and difficult to control exactly in paper-based devices with multiple sources of liquids and no hydrophobic confinement through patterning. The three frames represent 3 different points in time for the same experiment. As expected, the solution front and the associated dispersion zone advance more slowly as time goes on.

As the control experiment, paper devices with reagent channels but no further modification by hydrophobic patterning were produced. The paper devices were then subjected to co-flow mixing using the reaction between Fe^3+^ and SCN^−^ (Fig. 2). The appearance of a red band was observed at the interface between the two reagent solutions upon wetting of the paper by those reagent solutions. This means that transport of solutes occurred across the interface into the neighbouring solution. The area in which red colour is observed is thus the area in which mixing has occurred to some degree, due to transverse dispersion. We thus refer to the width of this band as the *dispersion zone*.

From Fig. 2, it is apparent that the flow rates for the two reagent solutions were not completely equal, which is a commonly encountered problem when working with paper microfluidics. In this case, the reagent channels were manually cut (guided by a line drawing), thus their sizes are likely to vary within a certain range (±1.5 mm). Furthermore, while the experimental setup was carefully controlled, the level and position of the reagent solution and reservoir, respectively, is not exactly identical, which could also lead to variation in capillary flow. The slight variation in capillary flow could be attributed to, firstly, the uneven floor of the glass chamber (in which the liquid reservoir being placed and paper being suspended) that could cause an uneven level of liquid despite of the identical volume of liquid and secondly, by the imperfectly vertical suspended paper.

When the width of the dispersion zone (red band; <3 mm) is compared to the width of the entire paper device (17 mm) through which the reagent solutions have travelled, the red band only occupies a fraction of that area. The reaction occurred only close to the interface between reagent streams. Clearly, the mixing between the two streams remained incomplete even after travelling for a distance of 45 mm. The long distance that the analytes need to travel transversely to the fluid front before being able to react is thus the main reason that co-flow mixing is ineffective in wider paper strips.

### 3.2 Co-flow mixing with the hourglass structure

One of the factors affecting mixing efficiency in wider strips of paper is the relative thinness of the paper. While the tortuosity of the porous network does enhance dispersion, when compared to unobstructed plug flow, the height across which dispersion can occur (equivalent to the paper thickness) is still limited. As a result, the contact area between the streams is also limited. As this geometric property is inherent to paper, it cannot readily be altered. Therefore, the width of the flow channel must be decreased to improve mixing efficiency.

**Fig. 3:**
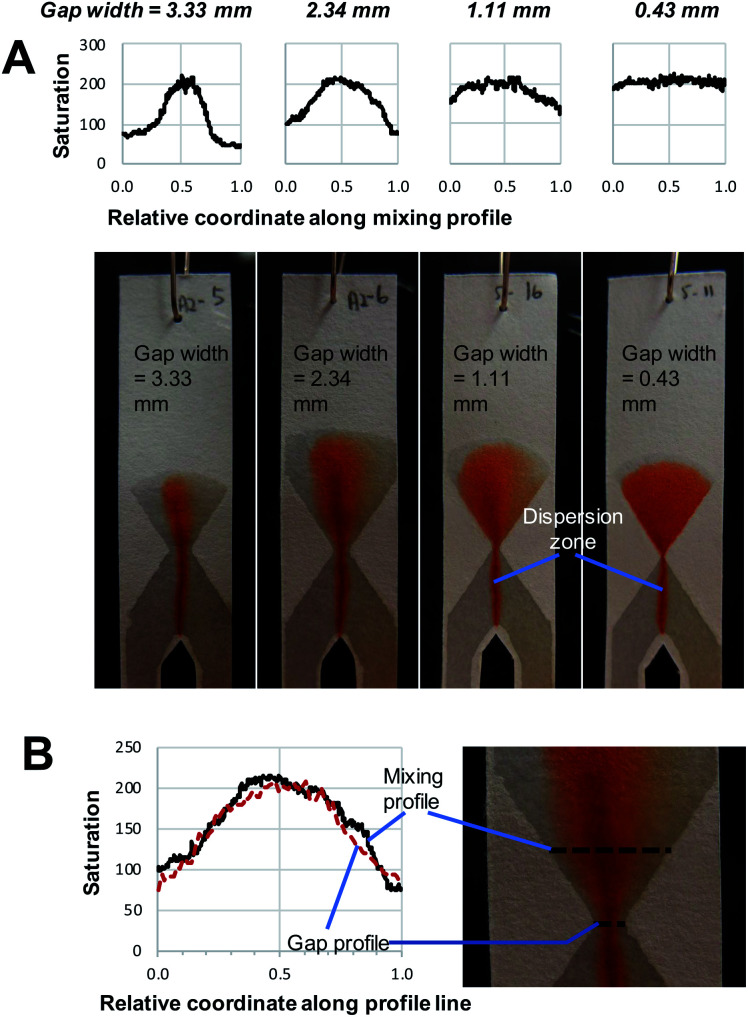
(A) Photographs show the mixing and product formation in paper devices using an hourglass structure with varying gap width. The respective plots of saturation values across the exit funnel at half-height are also given. These plots level off as gap width decreases, indicating enhanced mixing as gaps become smaller. (B) The blown-up image of an hourglass structure with a gap width of 2.34 mm, in which the saturation plots along the black lines highlights the similarity of the compositional profile across the gap (*i.e.* along gap profile) and the exit funnel (*i.e.* along mixing profile).

We thus introduced a flow restrictor, in the form of an hourglass structure as shown in Fig. 1 and 3, to reduce the channel width. This led to more effective mixing as can be seen by the wider spread of red colour of reaction product. This finding is in agreement with recent work by Schaumburg *et al.*, who applied a similar geometry to realize concentration gradient generators.^24^ Using such a structure, all liquid is forced to pass through a small gap or channel. If the gap is small enough, the limitation in transverse dispersion will not be significant enough to affect the total mixing, as liquids will be transported across the complete gap. The hourglass structure allows collection of all solutions introduced to the paper device, essentially focusing the streams and avoiding the creation of dead volumes. This alleviates the problem of variation of individual reagent solution flow rates that are the cause of the deviation of the dispersion zone from the centre of the device, as shown in Fig. 2. It does not, however, result in identical flow rates through both reagent channels.

The implementation of the hourglass structure yields a number of interesting results. First of all, the behaviour observed before the gap is similar to that seen in Fig. 2, where no hourglass structure was employed. That means that the position of the red dispersion zone shows deviation from the central vertical axis of the entrance funnel. However, as seen in Fig. 3, the hourglass structure largely rectifies this asymmetry. In addition, the use of an expanding exit funnel leads to increased spreading of the dispersion zone (the red band becomes broader), across the width of the device. Note that the horizontal mixing profile along the exit funnel is the expanded version of the profile along the gap (gap profile shown in Fig. 3B). Therefore, it is important to ensure that complete mixing is achieved at the gap.

#### Influence of the gap width

The gap width of the hourglass structure was varied in order to observe the effect of this parameter on the mixing performance. To better characterize how mixing proceeds in these devices, the pixel colour saturation across the gap and exit funnel was sampled and plotted.

**Fig. 4:**
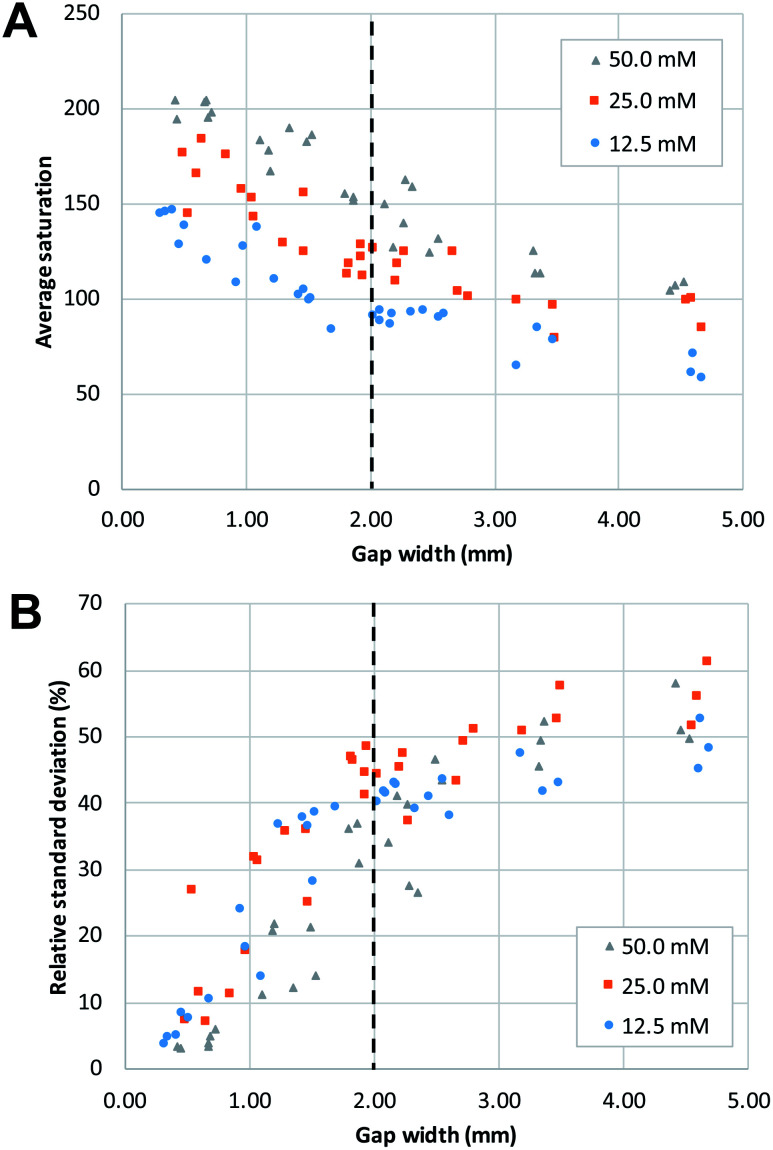
The effect of the gap width of the mixing structure on (A) average saturation and (B) relative standard deviation. Both reagents had the same concentration for any given mixing experiment. The dashed lines show the approximate gap width at which a change of trend can be seen.

By comparing the images and saturation plots in Fig. 3, we can see that smaller gaps lead on average to more intense, homogeneous red colour (high saturation across the gap and exit funnel) upon mixing, which indicates that more product was produced (Fig. 3A). We also observe that the smaller the gap, the more homogeneous the colour distribution (less variation of saturation) across the exit funnel. Below a certain gap size, the hourglass structure causes the exit funnel to be homogeneously filled with an intense red colour, which is indicative of complete mixing and relatively high reaction yields. This observation is in line with the hypothesis upon which development of this hourglass structure is based, namely that narrow flow channels facilitate mixing. However, the question of how small a gap should be could not be determined from this observation alone. The gap size of 1.11 mm, for example, seems well below the width of the initial dispersion zone (red band in the entrance funnel). Still, while the mixing appears to be good, it is not complete, as the varying saturation values across the exit funnel shows. This might be explained by the flow focusing effect of the hourglass structure. Being able to identify or predict the “cut-off point” for gap width below which excellent mixing can be obtained is important for the proper design of paper-based devices. Therefore, the effect of gap width on the average saturation and RSD of the mixing profile in the exit funnel was further studied using quantitative image analysis at three different reagent concentrations (Fig. 4).

In agreement with the visual observation, the quantitative image analysis showed that as the gap width becomes smaller, the average saturation of the defined area, thus the amount of product formed, increases (Fig. 4A). This increase becomes steeper as gap size decreases below 2 mm. This indicates that below this value, gap width has more effect on the reaction yield. The smallest gap size tested in this work (0.25 mm) was the smallest gap that we were able to produce that still allowed liquids to flow through. Potentially, with advances in patterning strategies and resolution, even smaller gap sizes could be produced with high reproducibility.

The effect of gap width on the RSD of mixing profiles such as shown in Fig. 3 is shown in Fig. 4B. Notably, the RSD becomes smaller as the gap width decreases. In other words, the mixing performance improves as gap widths get smaller. Again, we note that the reduction in RSD becomes more obvious starting at a gap width of *ca.* 2 mm. For widths above this value, the added value of the gap for the mixing performance is much less. It is also important to note that the reduction of RSD corresponds to a more constant saturation plot along the mixing profile, such as demonstrated in Fig. 3A by the paper device with gap of 0.43 mm in comparison to the other three larger-gap paper devices. This essentially means that the product is more homogeneously distributed across the exit funnel, and, if desired, channel branching for subsequent microfluidic processing could be performed at any location across the exit funnel.

As a positive control, a premixed solution of both reagents at 50 mM was allowed to wick into a strip of paper (*n* = 3). Image analysis was performed on these strips, and the obtained values (average saturation = 199) serve as reference for complete mixing, and maximum reaction yield (see ESI Fig. S2†). When these values are compared to the graphs in Fig. 4, we can conclude that complete mixing is obtained at values of the gap width of 0.5 mm or smaller. This means that there is a window of gap widths over which a gradient of mixing enhancement is observed, from about 0.5 mm (complete mixing) to about 2 mm, above which mixing enhancement ceases to be observed. This range of gap widths shall be referred to as the *transition window*.

**Fig. 5:**
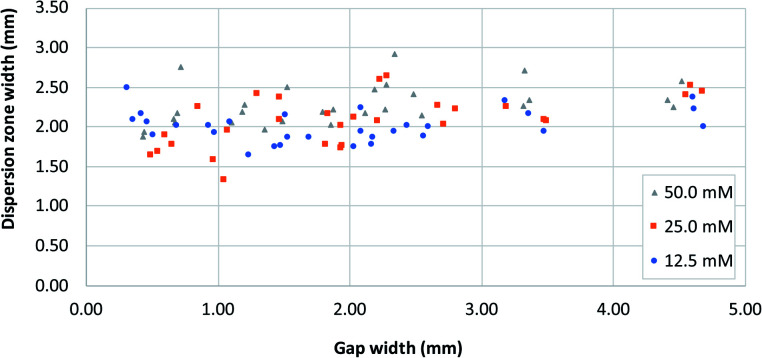
A plot of dispersion zone width before the gap (*i.e.* at dispersion profile line, Fig. 1B) in systems with different gap widths, for three different reagent concentrations. [Fe^2+^] and [SCN^−^] were equal in all experiments.

Fig. 5 shows the distribution of the transverse dispersion zone width in relation to the gap width of the same device. Fig. 5 demonstrates that the presence and size of the gap have no substantial influence on the width of the dispersion zone prior to reaching the gap. The average dispersion zone width is 2.11 mm (RSD = 13.57%) which corresponds to the same value that was found as the upper limit of the transition window. This means that when designing mixing structures, the width of the dispersion zone should be considered, which is dependent on the paper used, and the gap width should be designed to be smaller than the width of the dispersion zone.

However, as noted above, even at gap widths smaller than the dispersion zone width, sub-optimal mixing is obtained, as evidenced by the existence of the transition window in Fig. 4A. The reason for this is that the dispersion zone itself is not homogeneous across the gap width. This can be seen in Fig. 3B, where the red colour is most intense in the centre of the band, and where the mixing intensity profile exhibits a saturated (flattened) peak shape. Ideally, the gap width should not be larger than the width over which peak-shape is a maximum, which corresponds to approximately 0.5 mm. Having said that, the drawbacks of structures with small gaps must also be acknowledged. This includes (i) the long time needed for the liquid to fully wet the paper due to the low flow rate and (ii) the limits of the resolution of the patterning technique that might affect reproducibility.

#### Influence of reagent concentration

Different reagent concentrations were tested, as shown in Fig. 4 and 5. Higher concentrations of reagent produce higher average saturation which is of course expected as more product is formed. However, the main reason for testing different concentrations was to assess to which extent that would influence the width of the dispersion zone before the gap, and as a result the minimal gap width that would be necessary for obtaining complete mixing. Obviously, a larger concentration gradient leads to a higher rate of diffusion, which potentially could influence the width of the dispersion zone. However, no apparent differences are observed in the upper and lower limit of the transition window (Fig. 4B), nor in the width of the initial dispersion zone (Fig. 5). These findings are in agreement with previous research,^24^ which concluded that it is mainly mechanical dispersion that determines the width of the dispersion zone, determined by the structural properties of the porous cellulose network.

#### Influence of exit funnel angle

In addition to the gap size, the effect of the hourglass exit funnel angle on mixing performance was also tested (67, 30 and 15°). However, variations of this parameter were not found to affect the average saturation and RSD values. Plots of saturation for different exit angles can be found in the ESI, Fig. S3.† While saturation and RSD are not influenced by changing the exit funnel angle, variation in this parameter still will influence the system in terms of the linear flow rate after the solutions have reached the gap. The fluid front travels faster in an exit funnel with a smaller angle. This is due to the fact that an exit funnel with a bigger exit angle have a wider area (the length of the funnel was kept constant when angle was varied) thus allow for bigger volume of liquid to be adsorbed.^40^ A wider angle might contribute to the higher the volumetric flow rate though the gap due to the higher capillary pressure in the wider area. However, in depth analysis of this parameter as a result of geometrical variations was kept outside the scope of the current study.

### 3.3 Control of reagent stoichiometry for paper microfluidics

After optimization of its exact geometry as described above, the hourglass structure was also tested to see whether it could be used to control the composition of the mixture at and beyond the gap. This was done by changing the ratio between the width of the two reagent channels. To better visualize the composition change, a different chemical test model was used. The first reagent channel of the test paper was dipped in a 10 mM HCl solution containing Phenol Red (as pH indicator) while the second reagent channel was dipped in a 10 mM NaOH solution (without Phenol Red). At pH 6.4 and below, the colour of a solution containing the pH indicator will appear yellow, between pH 6.4 and 8.2 a red colour will appear, while at pH 8.2 and above, the colour will appear fuchsia.

**Fig. 6:**
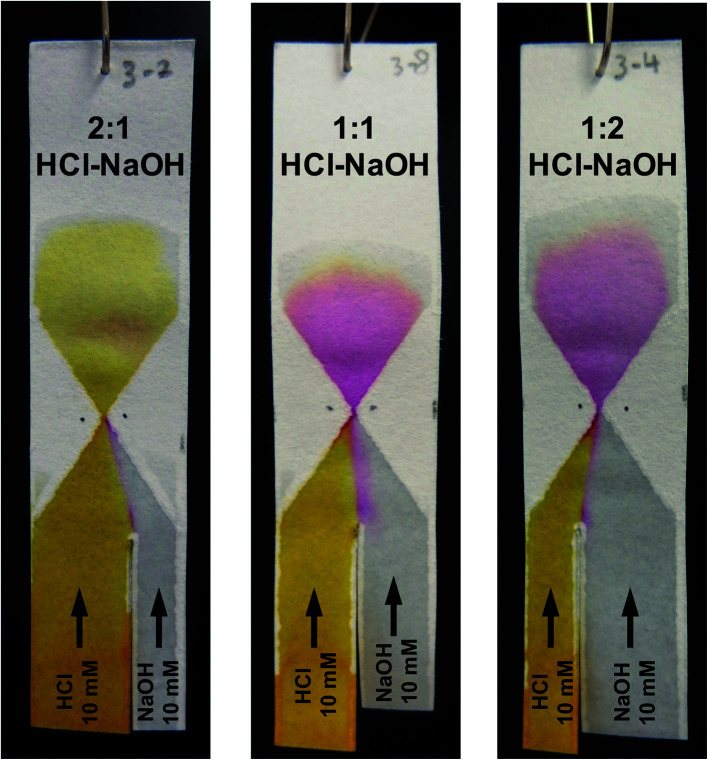
The effect of varying the reagent channel width ratio on the composition of the mixture. The left reagent channel was suspended in 10 mM HCl solution, containing 2 mM of Phenol Red. The right reagent channel was suspended in a 10 mM NaOH solution. The width of these reagent channels was varied in order to change the composition of the mixture beyond the hourglass structure. As the contributions of each solution vary, so does the final pH of the mixture, as evidenced by the different colours exhibited by the Phenol Red, a pH-sensitive dye. Essentially, the gap widths of these hourglass structures are <0.5 mm. The HCl and NaOH channel widths for 2 : 1, 1 : 1 and 1 : 2 are approx. 10 and 5 mm; 8 and 8 mm; and 5 and 10 mm, respectively.

As can be seen in Fig. 6, when the width ratio of HCl to NaOH is 2 : 1, the solution exhibited a yellow colour upon mixing. However, as the width ratio was changed to 1 : 1 and 1 : 2, the fuchsia colour was observed instead. This is in agreement with experiments done using premixed solutions of NaOH and HCl (data shown in Fig. S4†). The fuchsia colour appeared at the interface between the NaOH and HCl flow in the entrance funnel demonstrating the transverse dispersion across the interface.

The difference in the resulting colour is obvious between the 2 : 1 and 1 : 2 HCl–NaOH ratios. However, exact control over reaction stoichiometry has not been achieved in the experiments shown in Fig. 6, as the use of the hourglass paper microfluidic device still suffers from variation in the exact flowrates through each of the reagent channels, as alluded to in Section 3.1. Obtaining absolute control over these flowrates is an entirely different engineering challenge, and is beyond the scope of this work. Importantly, though, this experiment demonstrates the potential of obtaining complete mixing with the hourglass mixer, for a high degree of control over solution composition in paper microfluidics.

This approach can thus be used to define the composition of reaction mixtures on paper microfluidic devices, especially for reactions in which the stoichiometry is essential. Furthermore, it allows facile experimenting with different compositions of a reaction mixture (such as pH, ionic strength, reagent stoichiometry), without having to prepare individual solutions for each of them. Importantly, to be able to control the composition of a mixture in such a way, one needs to have good control over the mixing performance. Moreover, good mixing control will lead to reproducible reaction or dilution. Therefore, the implementation of a passive mixer such as described in this work is crucial.

## 4 Conclusions

The co-flow mixing of liquids on paper is minimal, limited by the extent to which transverse dispersion occurs. The degree to which mixing occurs is improved substantially by forcing the liquids to flow through a small gap. When this gap is no larger than the width of the dispersion zone, enhanced mixing is observed. Therefore, the gap width should be selected based on the dispersion zone width in order to maximize the mixing performance of the hourglass structure. However, to obtain truly complete mixing, the gap width should not be larger than the central region of the dispersion zone. This, however, is limited by fabrication methods for hydrophobic barriers. Also, small gap widths limit the flow rates through these gaps, which is often unwanted as it could increase assay times. Potential solutions to this, as have been suggested by others in previous work, are to use 3D paper microfluidics, which allows a larger interfacial area between two layers of paper, while the total thickness is limited to just the thickness of two sheets of paper. The latter is below the width of the gaps that were tested in this work. However, such a solution increases the complexity of paper microfluidic devices, which is undesirable when the end-goal is a consumer-friendly test.

Finally, by predefining the width ratio of the reagent channels, the hourglass mixer can be used to controllably alter the composition of a mixture (reaction stoichiometry) to be tailored according to the need of the developed assay. The proposed strategy thus serves as a simple and elegant approach to passively control and mix reagents in paper-based devices, to achieve good mixing and high reaction yields. Future work will focus on investigating the use of the developed mixers for doing quantitative chemical analysis in paper-based systems.

## Conflicts of interest

There are no conflicts to declare.

## Supplementary Material

RA-011-D1RA04916J-s001

## References

[cit1] Li H., Steckl A. J. (2019). Anal. Chem..

[cit2] Salentijn G. IJ., Grajewski M., Verpoorte E. (2018). Anal. Chem..

[cit3] Cate D. M., Adkins J. A., Mettakoonpitak J., Henry C. S. (2015). Anal. Chem..

[cit4] Morbioli G. G., Mazzu-Nascimento T., Stockton A. M., Carrilho E. (2017). Anal. Chim. Acta.

[cit5] Liana D. D., Raguse B., Gooding J. J., Chow E. (2012). Sensors.

[cit6] Kowkabany G. N., Cassidy H. G. (1952). Anal. Chem..

[cit7] Urteaga R., Elizalde E., Berli C. L. A. (2018). Analyst.

[cit8] Osborn J. L., Lutz B., Fu E., Kauffman P., Stevens D. Y., Yager P. (2010). Lab Chip.

[cit9] Hamidon N. N., Hong Y., Salentijn G. IJ., Verpoorte E. (2018). Anal. Chim. Acta.

[cit10] Fenton E. M., Mascarenas M. R., López G. P., Sibbett S. S. (2009). ACS Appl. Mater. Interfaces.

[cit11] Evans E., Moreira Gabriel E. F., Benavidez T. E., Tomazelli Coltro W. K., Garcia C. D. (2014). Analyst.

[cit12] Liu S., Su W., Ding X. (2016). Sensors.

[cit13] Xia Y., Si J., Li Z. (2016). Biosens. Bioelectron..

[cit14] Cummins B. M., Chinthapatla R., Lenin B., Ligler F. S., Walker G. M. (2017). Technology.

[cit15] Akyazi T., Tudor A., Diamond D., Basabe-Desmonts L., Florea L., Benito-Lopez F. (2018). Sens. Actuators, B.

[cit16] Rosenfeld T., Bercovici M. (2019). Lab Chip.

[cit17] Li X., Zwanenburg P., Liu X. (2013). Lab Chip.

[cit18] Wang J., Li W., Ban L., Du W., Feng X., Liu B. F. (2018). Sens. Actuators, B.

[cit19] Salentijn G. IJ., Hamidon N. N., Verpoorte E. (2016). Lab Chip.

[cit20] Akyazi T., Gil-González N., Basabe-Desmonts L., Castaño E., Morant-Miñana M. C., Benito-Lopez F. (2017). Sens. Actuators, B.

[cit21] Fratzl M., Chang B. S., Oyola-Reynoso S., Blaire G., Delshadi S., Devillers T., Ward T., Dempsey N. M., Bloch J. F., Thuo M. M. (2018). ACS Omega.

[cit22] Chen C., Zhao L., Zhang H., Shen X., Zhu Y., Chen H. (2019). Anal. Chem..

[cit23] Hong B., Xue P., Wu Y., Bao J., Chuah Y. J., Kang Y. (2016). Biomed. Microdevices.

[cit24] Schaumburg F., Urteaga R., Kler P. A., Berli C. L. A. (2018). J. Chromatogr. A.

[cit25] Ghosh R., Gopalakrishnan S., Savitha R., Renganathan T., Pushpavanam S. (2019). Sci. Rep..

[cit26] Carrell C., Kava A., Nguyen M., Menger R., Munshi Z., Call Z., Nussbaum M., Henry C. (2019). Microelectron. Eng..

[cit27] Fu E., Ramsey S. A., Kauffman P., Lutz B., Yager P. (2011). Microfluid. Nanofluidics.

[cit28] Masoodi R., Pillai K. M. (2010). AIChE J..

[cit29] MacDonald B. D. (2018). J. Fluid Mech..

[cit30] Bear J., Dynamics of Fluids in Porous Media, American Elsevier Publishing Company, New York, NY, 1972

[cit31] Murphy T. W., Zhang Q., Naler L. B., Ma S., Lu C. (2018). Analyst.

[cit32] Streets A. M., Huang Y. (2013). Biomicrofluidics.

[cit33] Nilghaz A., Zhang L., Shen W. (2015). Chem. Eng. Sci..

[cit34] Bhakta S. A., Borba R., Taba M., Garcia C. D., Carrilho E. (2014). Anal. Chim. Acta.

[cit35] Salentijn G. IJ., Grajewski M., Verpoorte E. (2017). Lab Chip.

[cit36] Rezk A. R., Qi A., Friend J. R., Li W. H., Yeo L. Y. (2012). Lab Chip.

[cit37] Cobb C. L., Love G. A. (1998). J. Chem. Educ..

[cit38] Nyasulu F., Barlag R. (2011). J. Chem. Educ..

[cit39] Schneider C. A., Rasband W. S., Eliceiri K. W. (2012). Nat. Methods.

[cit40] Elizalde E., Urteaga R., Berli C. L. A. (2015). Lab Chip.

