# Supplementary material for: Enhanced passive mixing for paper microfluidics
Source: RSC Adv. 2021 Jul 26;11(41):25677–85. doi: 10.1039/d1ra04916j (PMC8311897; doi:10.1039/d1ra04916j)
Supplement: RA-011-D1RA04916J-s001 [file RA-011-D1RA04916J-s001.pdf]

Electronic Supplementary Material (ESI) for RSC Advances.
This journal is © The Royal Society of Chemistry 2021

ELECTRONIC SUPPLEMENTARY INFORMATION

CO-FLOW MIXING ON PAPER-BASED DEVICES

Nurul Nadiah Hamidon [a, b], Gert IJ. Salentijn [c], Elisabeth Verpoorte [a,*]

[a] Pharmaceutical Analysis, Groningen Research Institute of Pharmacy, University of Groningen, 9700 AD Groningen, THE NETHERLANDS
[b] Faculty of Industrial Sciences and Technology, Universiti Malaysia Pahang, 26300 Kuantan, MALAYSIA
[c] Laboratory of Organic Chemistry, Wageningen University and Research, Stippeneng 4, 6708 WE Wageningen, THE NETHERLANDS
** Corresponding author*

TABLE OF CONTENTS

Step-by-step procedure for image analysis using ImageJ…..……S-2

Figures……………………………………………………..…………S-7

Figure S1: Microscopic image of the hourglass design……….......S-7
Figure S2: Saturation signal for KSCN-$FeCl_3$ solutions mixed…...S-7 off-paper
Figure S3: Influence of exit-funnel angle on mixing……………....S-8
Figure S4: Photographs of NaOH-HCl mixtures wicked into……..S-9 unpatterned paper strips

STEP-BY-STEP PROCEDURE FOR IMAGE ANALYSIS USING IMAGEJ

1. Open image in ImageJ

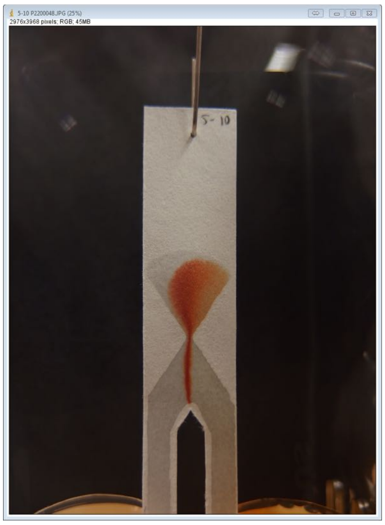

2. Assign region of interest (ROI). Save the ROIs as “**ROI Set A**” for later reference

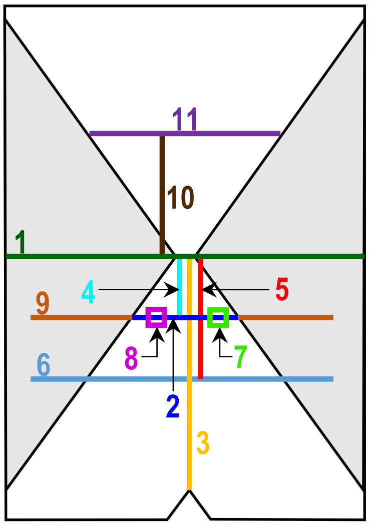

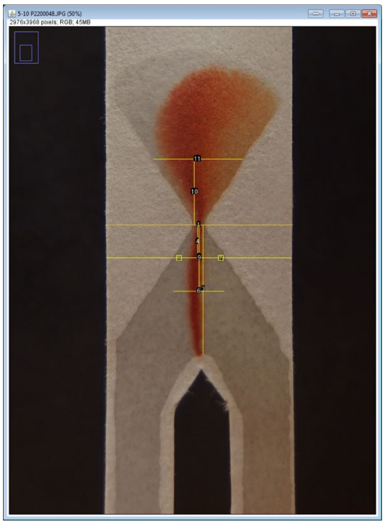

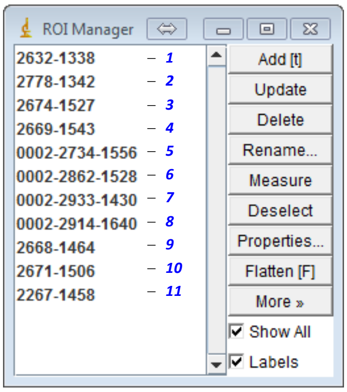

ROI 1: The width of paper at the height of the gap.

ROI 2: Dispersion profile. Assigned at the same level of ROI 9.

ROI 3: The length of the entrance funnel.

ROI 4: Half the length of ROI 5 / Quarter the length of ROI 3.

ROI 5: Half the length of ROI 3.

ROI 6: Reference mark for half the length of the entrance funnel.

ROI 7: A square reference area for sampling KSCN baseline signal.

ROI 8: A square reference area for sampling $FeCl_3$ baseline signal.

ROI 9: Reference mark for quarter the length of the entrance funnel.

ROI 10: Half the length of ROI 3.

ROI 11: Mixing profile. Assigned at the same distance.

(The ROIs were assigned on the image according to the following order:

1 → 3 → 6 → 5 → 9 → 4 → 10 → 11 → 8 → 7)

3. Convert image to HSB stacks

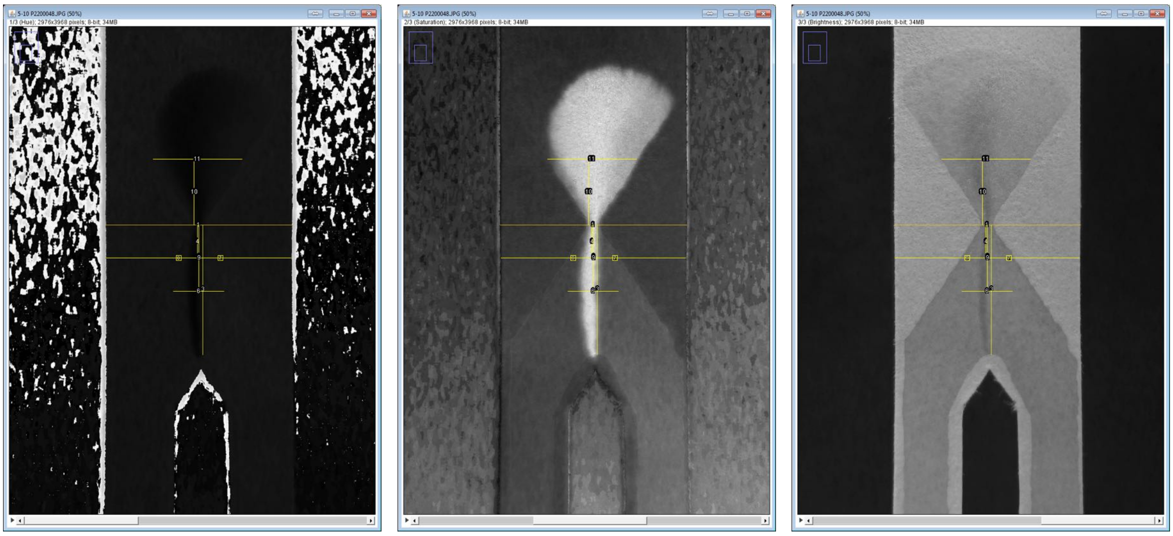

4. Perform ROIs measurement, save into Excel file

Results

File Edit Font Results

	Label	Area	Mean	StdDev	Min	Max	Angle	Length
1	5-10 P2200048.JPG:2632-1338:Saturation	760	63.817105263	29.621938813	10	219	0	759
2	5-10 P2200048.JPG:2778-1342:Saturation	205	116.131707317	53.765989136	60	219	0	204
3	5-10 P2200048.JPG:2674-1527:Saturation	528	144.560606061	13.784741214	106	178	-90	527
4	5-10 P2200048.JPG:2669-1543:Saturation	134	205.746268657	12.498232397	164	231	-90	133.333251953
5	5-10 P2200048.JPG:0002-2734-1556:Saturation	264	210.996212121	9.796988016	184	245	-90	263.333251953
6	5-10 P2200048.JPG:0002-2862-1528:Saturation	207	114.067632850	51.631397704	64	222	0	206.000000000
7	5-10 P2200048.JPG:0002-2933-1430:Saturation	400	75.992500000	3.764688027	66	96	0	0.000000000
8	5-10 P2200048.JPG:0002-2914-1640:Saturation	400	64.130000000	2.608661109	57	74	0	0.000000000
9	5-10 P2200048.JPG:2668-1464:Saturation	754	73.468169761	38.462098004	23	219	0	753.000000000
10	5-10 P2200048.JPG:2671-1506:Saturation	270	195.696296296	24.332967242	116	229	90	269.333251953
11	5-10 P2200048.JPG:2267-1458:Saturation	362	178.129834254	37.294920220	60	227	0	361.333374023

5. Choose the ROI for mixing profile (ROI 11)

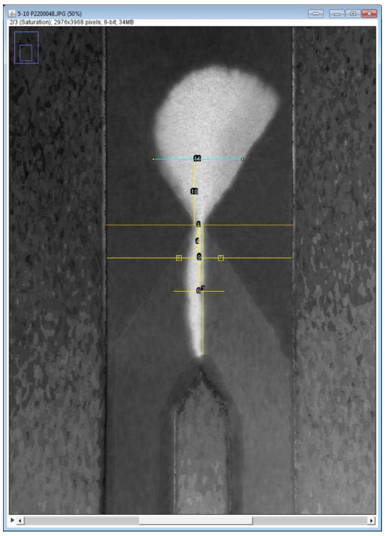

6. Generate the Plot Profile for the ROI of the mixing profile line, save the plot value into Excel file (to be used later for calculating average saturation and standard deviation).

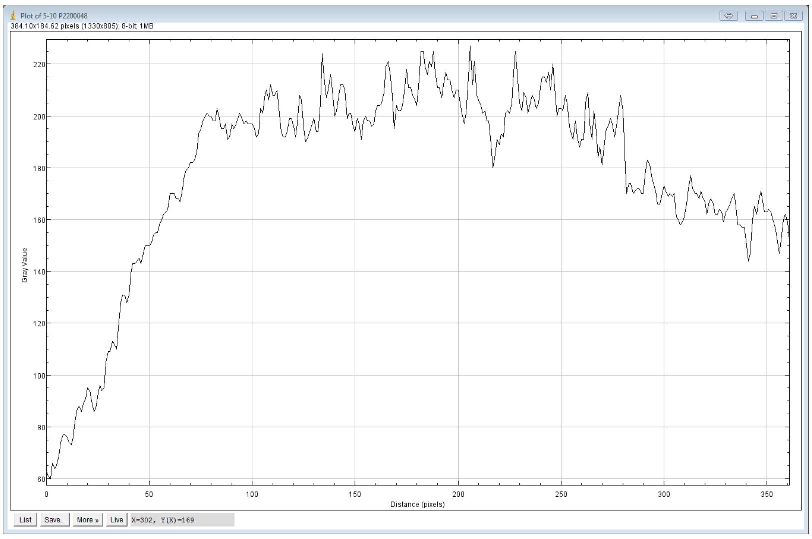

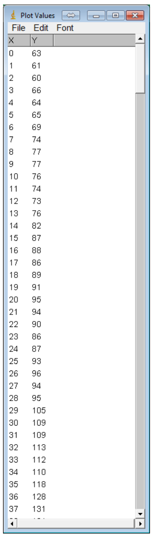

7. Choose the ROI for a horizontal line which is ¼ of the distance from the gap (ROI 2)

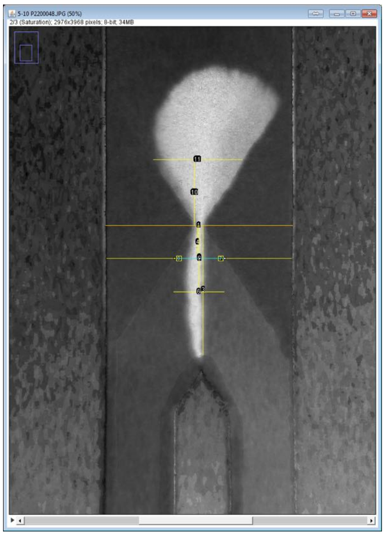

8. Generate the Plot Profile of the selected ROI

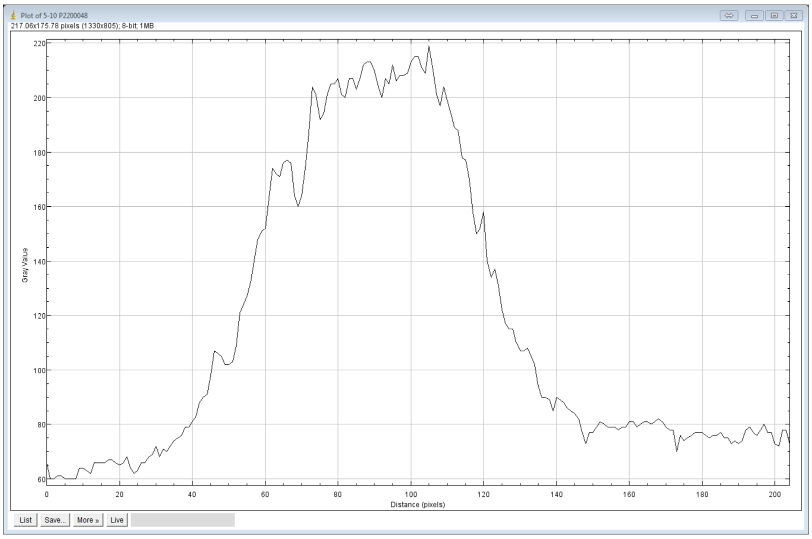

9. Assign ROIs. ROIs were saved as “**ROI Set B**” for later reference

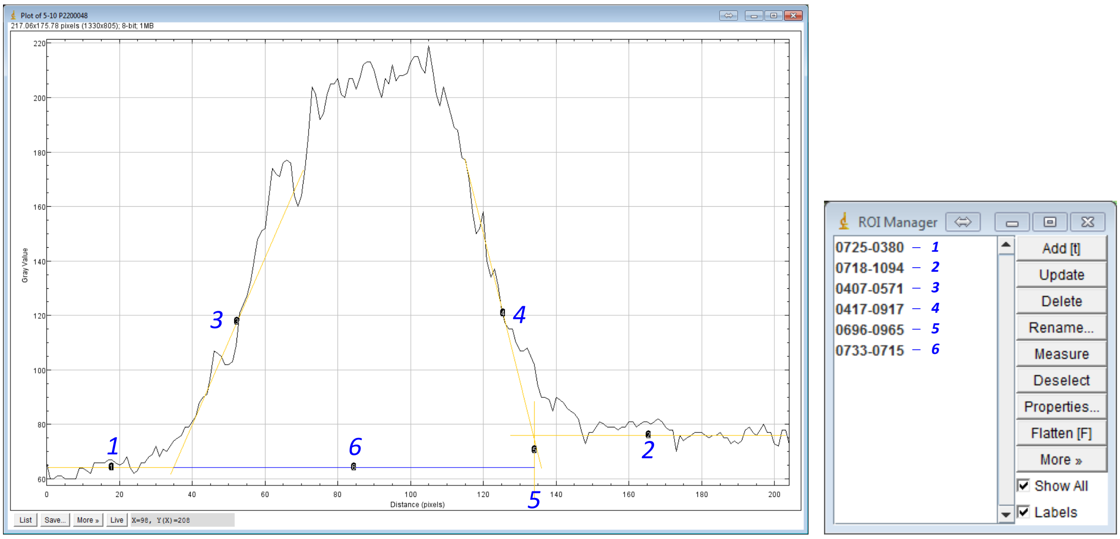

ROI 1: The background saturation of KSCN, the value was obtained from the ROI 8 (average saturation) of the ROI Set A.

ROI 2: The background saturation of $FeCl_3$, the value was obtained from the ROI 7 (average saturation) of the ROI Set A.

ROI 3 & ROI 4: A tangent line to best represent the increment/decline of saturation (along Y-axis).

ROI 5: A vertical line representing the ROI 2-ROI 4 intercept (along X-axis).

ROI 6: A horizontal line stretching between the ROI 1-ROI 3 intercept and ROI 5. This line basically represents the “peak width” of the diffusion band. By taking consideration of the background saturation of the two reagents and estimated slope/tangent of as threshold.

FIGURES

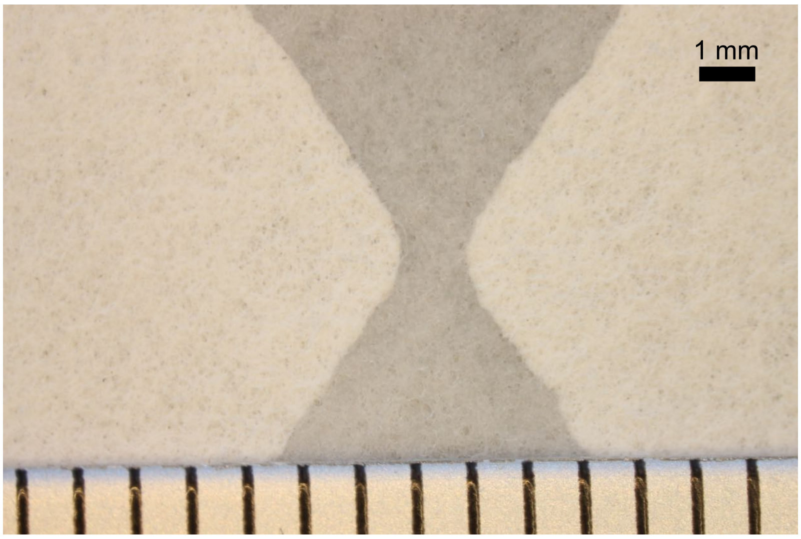

Figure S1: Image of an hourglass structure on paper wetted with water, acquired using microscope and camera.

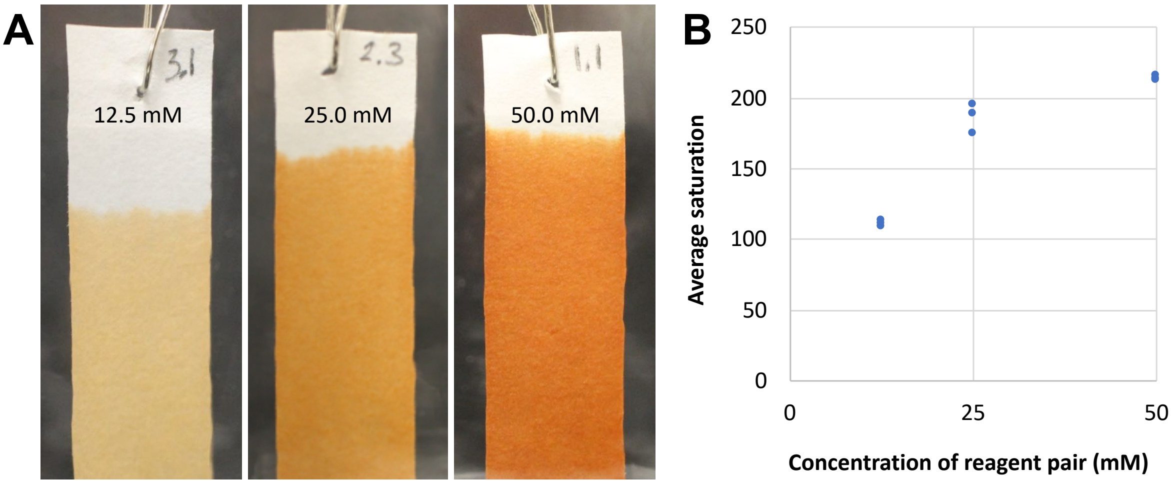

Figure S2: Saturation signal for KSCN-$FeCl_3$ solutions mixed off-paper. Three mixtures were prepared by mixing 1:1 ratio of KSCN solution and $FeCl_3$ solution having the same concentration; three different concentrations were used (12.5 mM, 25.0 mM and 50.0 mM). (A) One end of the untreated paper was dipped into the mixture to allow wicking into the paper. (B) A plot showing the average saturation of the pre-mixed solutions wicked into paper for the concentrations tested (*n* = 3 paper strips for each concentration).

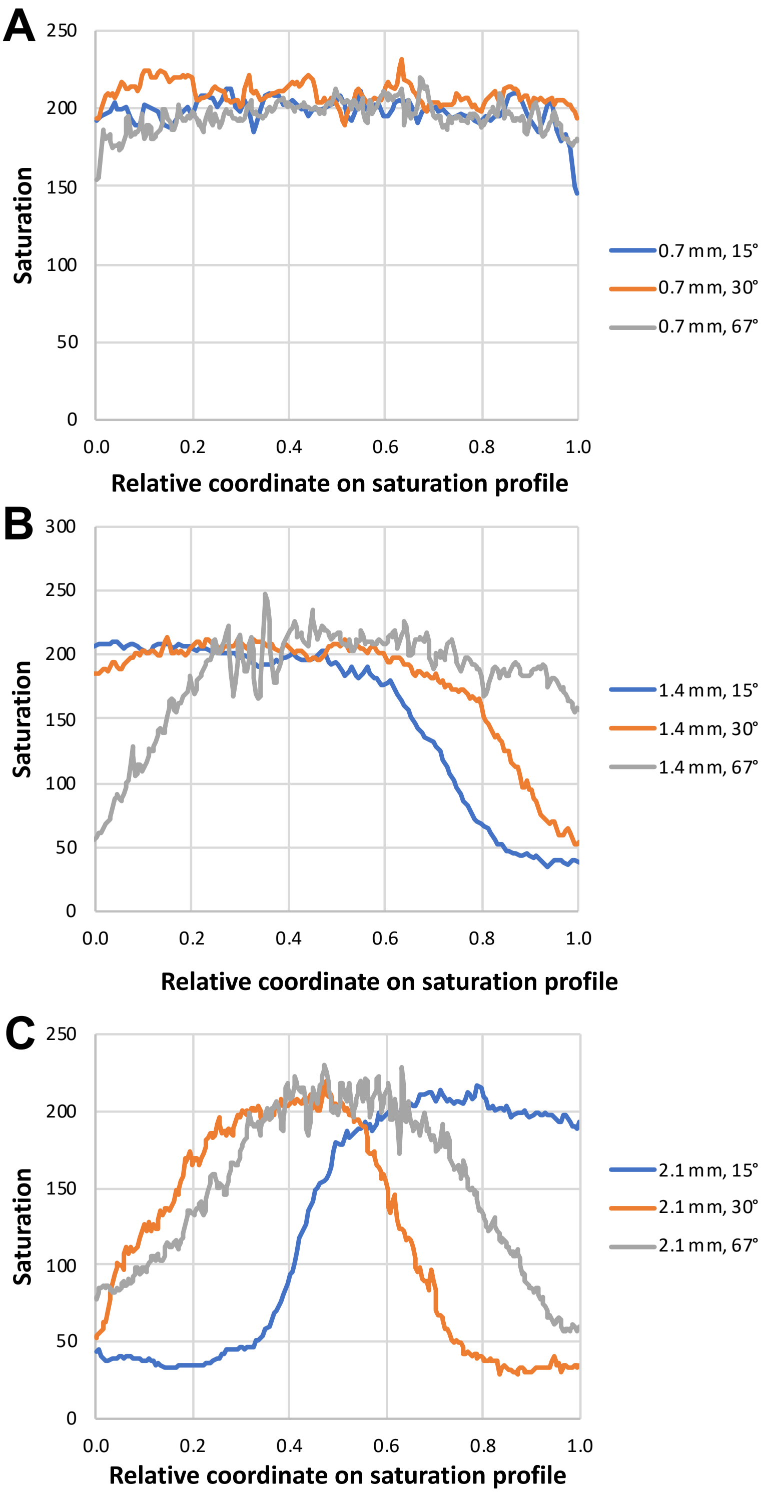

Figure S3: Saturation recorded along the mixing profile for three different exit-funnel angles for gap widths of ca. (A) 0.7 mm, (B) 1.4 mm and (C) 2.1 mm. It is apparent that good mixing can be obtained with small gaps regardless of the funnel angle. As the gap becomes bigger, steep changes in saturation are observed, indicating incomplete mixing.

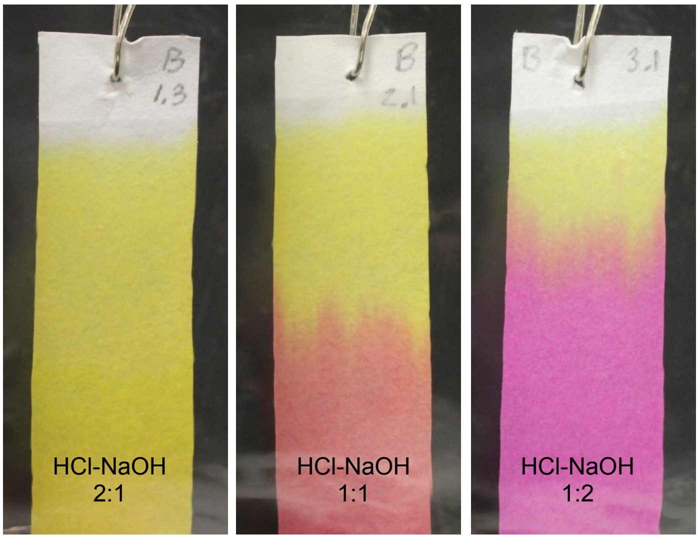

Figure S4: Photographs of HCl-NaOH mixtures wicked into unpatterned paper strips. Three mixtures were prepared by mixing 10 mM HCl solution containing Phenol Red (2 mM; as pH indicator) and 10 mM NaOH solution (without Phenol Red) at ratios of 2:1, 1:1 and 1:2. One end of the untreated paper strip was then dipped into the mixed solution to allow wicking into the paper.
